# Green tea halts progression of cardiac transthyretin amyloidosis: an observational report

**DOI:** 10.1007/s00392-012-0463-z

**Published:** 2012-05-15

**Authors:** Arnt V. Kristen, Stephanie Lehrke, Sebastian Buss, Derliz Mereles, Henning Steen, Philipp Ehlermann, Stefan Hardt, Evangelos Giannitsis, Rupert Schreiner, Uwe Haberkorn, Philipp A. Schnabel, Reinhold P. Linke, Christoph Röcken, Erich E. Wanker, Thomas J. Dengler, Klaus Altland, Hugo A. Katus

**Affiliations:** 1 Department of Cardiology, Angiology, and Respiratory Medicine, University Hospital Heidelberg, Im Neuenheimer Feld 410, 69120 Heidelberg, Germany; 2 Laboratory of Dr. Limbach and Associates, Im Breitspiel 15, 69126 Heidelberg, Germany; 3 Department of Nuclear Medicine, University of Heidelberg, Im Neuenheimer Feld 400, 69120 Heidelberg, Germany; 4 Institute of Pathology, Heidelberg University, Im Neuenheimer Feld 220/221, 69120 Heidelberg, Germany; 5 Reference Center of Amyloid Diseases, amYmed, Am Klopferspitz 19, 82152 Martinsried, Germany; 6 Institute of Pathology, Christian-Albrechts-University, Arnold-Heller-Straße 3/14, 24105 Kiel, Germany; 7 Max Delbrück Center for Molecular Medicine, AG Neuroproteomics, 13092 Berlin, Germany; 8 Institute of Human Genetics, Giessen University, Schlangenzahl 14, 35392 Giessen, Germany

**Keywords:** Cardiomyopathy, Green tea, Transthyretin-derived amyloidosis, Epigallocatechin-3-gallate

## Abstract

**Background:**

Treatment options in patients with amyloidotic transthyretin (ATTR) cardiomyopathy are limited. Epigallocatechin-3-gallate (EGCG), the most abundant catechin in green tea (GT), inhibits fibril formation from several amyloidogenic proteins in vitro. Thus, it might also halt progression of TTR amyloidosis. This is a single-center observational report on the effects of GT consumption in patients with ATTR cardiomopathy.

**Methods:**

19 patients with ATTR cardiomyopathy were evaluated by standard blood tests, echocardiography, and cardiac MRI (*n* = 9) before and after consumption of GT and/or green tea extracts (GTE) for 12 months.

**Results:**

Five patients were not followed up for reasons of death (*n* = 2), discontinuation of GT/GTE consumption (*n* = 2), and heart transplantation (*n* = 1). After 12 months no increase of left ventricular (LV) wall thickness and LV myocardial mass was observed by echocardiography. In the subgroup of patients evaluated by cardiac MRI a mean decrease of LV myocardial mass (−12.5 %) was detected in all patients. This was accompanied by an increase of mean mitral annular systolic velocity of 9 % in all 14 patients. Total cholesterol (191.9 ± 8.9 vs. 172.7 ± 9.4 mg/dL; *p* < 0.01) and LDL cholesterol (105.8 ± 7.6 vs. 89.5 ± 8.0 mg/dL; *p* < 0.01) decreased significantly during the observational period. No serious adverse effects were reported by any of the participants.

**Conclusions:**

Our observation suggests an inhibitory effect of GT and/or GTE on the progression of cardiac amyloidosis. We propose a randomized placebo-controlled investigation to confirm our observation.

**Electronic supplementary material:**

The online version of this article (doi:10.1007/s00392-012-0463-z) contains supplementary material, which is available to authorized users.

## Introduction

Cardiac ATTR amyloidosis is observed as the predominant manifestation associated with distinct mutations in the TTR gene also known as familial amyloid cardiomyopathy (FAC) [1] and represents the major complication in patients of advanced age with familial amyloid polyneuropathy (FAP) [2]. Wild-type TTR (senile systemic amyloidosis, SSA) has been found in 25 % of post-mortem cardiac biopsies from patients older than 85 years [3]. Pre-mortem diagnosis is rare, but prevalence appears to be underestimated. By routine scintigraphic skeletal imaging SSA has been detected in 2 males among 374 consecutive admissions older than 60 years, indicating a prevalence of about 0.5 % [4].

Without treatment both hereditary and non-hereditary forms of cardiac ATTR amyloidosis are progressing and fatal after several years. Patient’s prognosis mostly depends on the extent of cardiac involvement. Despite that therapeutic options for patients with light-chain amyloidosis have been ameliorated during the past years [5], treatment concepts for patients with advanced cardiac involvement in TTR amyloidosis are still limited. Liver transplantation has become an accepted causative treatment to stop the hepatic production of variant TTR [6] in FAP and FAC patients, but progression of cardiac amyloid deposition has been observed rather frequently after liver transplantation [7]. Patients with advanced cardiac amyloidosis are deemed ineligible for this approach due to high treatment-related mortality. Liver transplant has no therapeutic effect in SSA patients. Heart transplantation remains the only therapeutic option for advanced cardiac TTR amyloidosis but is limited to patients at age <65 years.

Assuming a prevalence of 0.5 % for clinically relevant cardiac ATTR amyloidosis in the growing elderly population (>60 years) there is a high need for novel effective preventive and therapeutic measures against this otherwise fatal disease. Experimental studies evaluated the suppression of hepatic synthesis of TTR by antisense oligonucleotids [8]. Several small compounds, e.g. non-steroidal anti-inflammatory drugs or derivatives [9], flavonoids [10], xanthones of plant origin [11], and sulfite [12] have been suggested for treatment of ATTR amyloidosis by inhibiting TTR tetramer dissociation and/or impairing amyloid fibril formation. Recently, Tafamidis (Fx-1006A), a strong inhibitor of TTR tetramer dissociation has been approved for the treatment of patients with FAP stage I (http://www.ema.europa.eu/ema/index.jsp?curl=pages/medicines/human/medicines/002294/human_med_001498.jsp&mid=WC0b01ac058001d124).

Laboratory studies have shown that human TTR is rather stable at physiological pH 7.4 but tends to denature at pH 6.5–7, levels observed under inflammatory or ischemic conditions, indicating that prevention of such conditions could help to reduce the risk for TTR to denature and become converted into amyloid [13]. Recent in vitro experiments have shown that 50 μM epigallocatechin-3-gallate (EGCG), the most abundant catechin in green tea (GT), efficiently inhibits fibril formation from amyloid ß-protein, α-synucleine [14], and TTR [15] and converts existing fibrils into non-fibril conformers [16]. In a FAP transgenic mouse model a reduction of amyloid deposition was observed after treatment with 100 mg EGCG per kg body weight per day over a period of 6 weeks [17]. A retired member of our faculty (Dr. W. Hunstein), observed a 25 % decrease of the LV wall thickness after having consumed 1.5–2 L of GT daily over 11 months [18, 19]. According to manufacturer information the green tea used by Hunstein contained about 350 mg EGCG per liter when brewed for 5 min at 100 °C. A decrease of LV wall thickness >2 mm was observed in 11 AL amyloidosis patients with history of regular GT consumption [20]. The initial in vitro studies by Ehrnhoefer et al. [14] as well as the personal in vivo observation of Hunstein [18] stimulated our trial to reproduce Hunstein’s observation in patients with cardiac ATTR amyloidosis.

## Methods

The present cohort at the Heidelberg Amyloidosis Center consisted of 19 patients (15 males, 4 females; mean age 66.3 ± 2.0 years) with hereditary (*n* = 10, 53 %) or non-hereditary SSA (*n* = 9, 47 %) cardiac ATTR amyloidosis and compensated heart failure. Patients were observed over a period of 12 months while consuming GT and/or GTE. Routine medication was continued during this period. The observation was approved by the institutional Ethics Committee of the University of Heidelberg and complied with the Declaration of Helsinki [21].

**Fig. 1:**
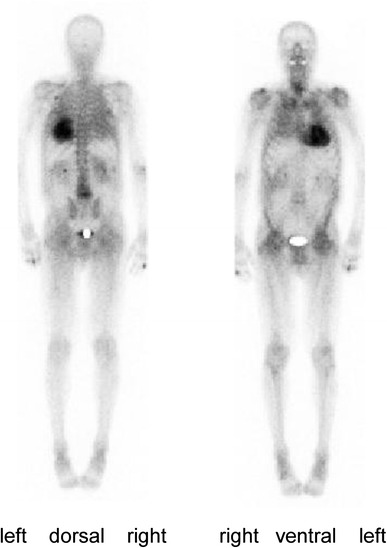
Whole body scintigraphy for visualization of cardiac transthyretin amyloid Cardiac tracer uptake obtained 3 h after application of 736 MBq technetium-99 m 3,3-diphosphono-1,2-propanodicarboxylic acid (^99m^Tc-DPD) indicating cardiac ATTR amyloid deposition

All patients had biopsy-proven ATTR confirmed by green birefringence of Congo red-stained sections viewed in polarized light and subsequent immunohistochemical classification of the amyloid deposits [22, 23]. They were screened for amyloidogenic TTR variants by isoelectric focusing of plasma TTR and by sequencing of genomic DNA [24]. Clonal light-chains were examined by serum/urine immunofixation electrophoresis and a serum-free light-chain test. Monoclonality was excluded in all patients. All patients had increased tracer retention in the heart by ^99m^Tc DPD scintigraphy (Fig. 1) indicating cardiac ATTR depositions [25, 26].

In addition to cardiac involvement patients with hereditary TTR amyloidosis had clinical evidence for involvement of gut (*n* = 4; 40 %), and/or peripheral nerves (*n* = 8; 80 %). The main clinical symptoms at study entry were polyneuropathy in six patients and exertional dyspoea in eight patients. Mean functional heart failure class according to New York Heart Association was 2.0 ± 0.2. Four patients (40 %) with hereditary amyloidosis had orthotopic liver transplantation prior to study inclusion.

After having received written informed consent for participation in this observational project the patients were evaluated by standard blood tests, echocardiography with Doppler studies, and cardiac magnetic resonance imaging (cMRI). Follow-up controls were performed by clinical investigation at the Heidelberg Amyloidosis Center 6 and 12 months after study inclusion.

### Echo- and electrocardiography

All transthoracic echocardiograms were performed according to a routine protocol using commercially available ultrasound diagnostic systems (Vivid 7, GE Healthcare, Milwaukee, WI, USA) by experienced investigators. Three cardiac cycles were stored in cine loop format for offline analysis. In patients who were in atrial fibrillation five consecutive beats were averaged. Offline analysis of echocardiography examinations of both cohorts were conducted on a commercially available workstation (Centricity Cardiology CA1000 2.0, GE Medical Systems, Milwaukee, WI, USA) independently by three expert investigators blinded by time point. M-mode or 2D recordings were analyzed for diastolic interventricular septum thickness (IVS), diastolic posterior wall thickness, end-diastolic and end-systolic left ventricular (LV) cavity diameter, LV systolic and longitudinal function, and pericardial effusion [27]. Left ventricular mass was calculated by the Devereux formula [28]. LV function was assessed according to standard definitions [29] and was considered markedly impaired at <40 % by the biplane Simpson method (2D/4D). LV longitudinal function was assessed by mitral and tricuspid annular plane systolic excursion from the apical 4-chamber view by measuring the maximum excursion of the lateral site of the annulus after ventricular systole by M-mode imaging. Pulsed-wave Doppler tissue imaging of the mitral annulus was done using a standard spectral Doppler sample volume gate length of 0.17 cm. The sample volume was placed on the lateral mitral annulus in the apical four-chamber view to measure systolic velocity.

### Cardiac magnetic resonance imaging

CMRI was performed on a 1.5-T whole body MRI scanner (Achieva Intera1 Philips Medical Systems, Best, The Netherlands) equipped with a five-element cardiac receiver coil according to a protocol that has been described in detail previously [30]. Resting LV function was determined by cine images using a segmented k-space balanced fast-field-echo sequence (Steady-State-Free-Precession, matrix = 160/256, sense-factor = 2, flip angle = 60°, slice thickness/gap = 8/2 mm) in contiguous short axes covering the whole left ventricle from base to apex as well as two, three, and chamber views in anatomically correct heart axes. Data analysis was carried out on a commercially available cMRI workstation (Philips Viewforum, Version 3.4, Best, The Netherlands) by three independent observers blinded by time point. LV enddiastolic and endsystolic volumes with resulting ejection fraction as well as myocardial mass in diastole were determined from short axes by manual delineation of endocardial and epicardial borders excluding papillary muscles.

### Dosage of green tea and green tea extract

The protocol followed that of Hunstein [18]. All patients were asked for a daily intake of approximately 500–700 mg EGCG either by drinking 1.5–2 L of GT per day (10 g/L Green Darjeeling, FTGFOP1, Teekampagne Projektwerkstatt GmbH, Berlin, Germany; brewed 3–5 min at 100 °C, and containing about 340 mg EGCG/L; [31]) or by the intake of caffeine-free GTE capsules (praevent-loges^®^, Dr. Loges + Co. GmbH, Winsen/Luhe, Germany, containing 300 mg GTE with 75 mg EGCG/capsule). Patients were asked to document in a protocol their daily GT and/or GTE consumption as well as any adverse effects. This protocol was used to calculate the mean daily EGCG intake during the observational period [(total GT (in L) ×340 mg/L × total number of GTE capsules ×75 mg)/days of observation].

### Quantification of plasma EGCG levels

Blood samples were collected in EDTA-containing tubes. As EGCG rapidly changes its structure above pH 7, binds to proteins, and becomes oxidized, the blood samples were immediately transferred to an ice bath and the plasma was separated from the cellular fraction by centrifugation at 4 °C. One ml of each plasma sample was mixed with 50 μL of an ascorbate-EDTA solution [0.4 M NaH_2_PO_4_ buffer containing 20 % ascorbic acid and 0.1 % EDTA (pH 3.6)] and stored at −20 °C until analysis. The EGCG levels were measured via LC-MSMS (liquid chromatography equipped with a triple quadruple mass spectrometer). In short, 100 μL of the stabilized plasma was acidified with 20 μL 5 N HCl and extracted twice with ethyl acetate. The organic phase was evaporated and resolved in 300 μL of a solution containing two volumes of a 20-mM ammonium acetate buffer pH 3.6 and 1 volume methanol. The separation was performed on a C18 Reversed-Phase Column with a gradient of 20 mM ammonium acetate against methanol. The transitions monitored in the mass selective detector under negative modus were 457 (precursor Ion) to 125 product Ion, 457 (precursor Ion) to 169 product Ion (qualifier), and as internal standard 319 (precursor Ion) to 191 product Ion.

### Statistical analysis

Continuous data were expressed as median and range. Categorical variables were expressed as absolute numbers and percentages. Differences between the non-parametric, continuous parameters assessed at study inclusion and at 12-month follow-up visit were compared by the Wilcoxon rank-sum test. *p* < 0.05 was considered statistically significant. Statistical analyses were performed using StatView (Version 5.0, SAS Institute, Cary, NC, USA).

## Results

### Patients

Of a total of 19 examined patients five patients were lost during the study period either due to death (*n* = 2), discontinuation of green tea consumption (*n* = 2), or heart transplantation (*n* = 1). All study patients (*n* = 14) were assessed by echocardiography. One patient died 1 month after study inclusion due to cardiac failure; the second death occurred after a 5-month stay at the intensive care unit with prolonged artificial ventilation, hemofiltration, recurrent infections, and finally death due to multi-organ failure after drainage of a retroperitoneal hematoma. Due to prior placement of cardiac pacemaker (*n* = 2), internal defibrillator (*n* = 1), orthopedic prosthesis (*n* = 1), or claustrophobia (*n* = 1) cMRI images were obtained only from 9 of the 14 study patients. The clinical state of the 14 study patients as well as oral heart failure medication remained unchanged during the observation period. None of the patients was hospitalized due to cardiac decompensation. The main clinical demographics of the 14 patients with 12-month follow-up are shown in Table 1. Detailed data for all 14 patients at the beginning and the end of the observation period are given in Table 1S (Supplemental information).

**Table 1:** Clinical demographics of the patients included in the study

Clinical demographics	All patients (*n* = 14)	Patients studied by cMRI (*n* = 9)
	Mean ± SEM or number (%)	Mean ± SEM or number (%)
Age (years)	66 ± 3	68 ± 2
Gender male/female	10 (71 %)/4 (29 %)	7 (78 %)/2 (22 %)
Height (cm)	169 ± 2	171 ± 2
Weight (kg)	71 ± 3	72 ± 3
Modified body mass index^a^	1,080 ± 46	1,084 ± 45
Patients with variant TTR	8 (57 %)	5 (56 %)
Val20Ile	0	0
Val30Met	5 (36 %)	4 (44 %)
Gly47Glu	1 (7 %)	0
Gly47Ala	1 (7 %)	0
Ile107Val	1 (7 %)	1 (12 %)
Patients with SSA	6 (43 %)	4 (44 %)
Orthotopic liver transplant	2	1
Pacemaker placement	2	–
Defibrillator placement	1	–
Orthopedic prosthesis	1	–

*FU* follow-up

^a^Body weight (kg)/body height (m^2^) × plasma albumin (d/L)

### Quantification of plasma EGCG levels, cholesterol, and NT-proBNP

**Fig. 2:**
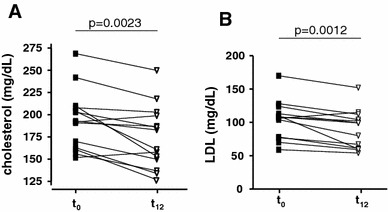
Changes of lipid profile during the study period Total cholesterol (**a**) and LDL cholesterol (**b**) plasma levels of patients with TTR amyloidosis before (*t*_0_) and after 12 months (*t*_12_) of green tea consumption

All patients documented their daily intake of GT/GTE. According to the protocols mean oral EGCG intake was calculated as 547 ± 49 mg per day. Individual mean daily EGCG intake is shown in the supplementary table. No serious adverse effects were reported. Mean EGCG plasma level after 12 h of GT/GTE abstinence was 48 ± 14 μg/L (0.1 ± 0.03 μM). Mean plasma EGCG level 2 h after intake of 150 mg EGCG as GTE (2 capsules) was 253 ± 68 μg/L (0.55 ± 0.15 μM, range 0.1–1.8 μM). During the observation period mean total cholesterol and LDL cholesterol decreased from 191.9 ± 8.9 to 172.7 ± 9.4 mg/dL (*p* < 0.01) and from 105.8 ± 7.6 to 89.5 ± 8.0 mg/dL (*p* < 0.01), respectively (see Fig. 2a, b). Mean HDL cholesterol remained unchanged (58.9 ± 3.9 to 55.4 ± 4.6 mg/dL (*p* < 0.01).

### Echocardiography analysis

**Fig. 3:**
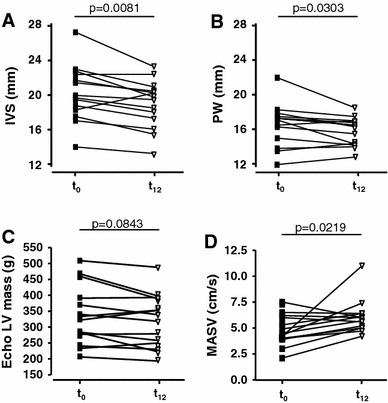
Echocardiographic changes during the study period (**a**) Interventricular septum thickness, (**b**) posterior wall thickness, (**c**) left ventricular myocardial mass, and (**d**) systolic velocity of the lateral mitral annulus (MASV) assessed by echocardiography in patients with TTR amyloidosis before (*t*_0_) and after 12 months (*t*_12_) of green tea consumption

During 12 months of GT/GTE intake a statistically significant decrease by 6.5 % of the thickness of IVS was observed (Fig. 3a). Thickness of IVS decreased in 12/14 patients (86 %); in 4 patients by ≥2 mm, remained unchanged in 1 patient (7 %) and increased 2 mm in 1 patient (7 %). Mean thickness of the posterior wall did not change during the study period (Fig. 3b). A decrease of the posterior wall thickness was observed in 10/14 patients (71 %; ≥2 mm in 2 patients), and an increase of less than 2 mm was detected in 4/14 patients (29 %). Mean LV myocardial mass did not increase during the observational period of GT/GTE intake (Fig. 3c). In detail, LV myocardial mass decreased in 9/14 (64 %) patients, increased in 3/14 (21 %) patients, and remained unchanged in 2/14 (14 %) patients. A significant improvement of the mean systolic velocity of the lateral mitral annulus was observed (improved in 10/14 patients (71 %); diminished in 4/14 (14 %) patients; Fig. 3d). More detailed echocardiographic data are shown in Table 1S (Supplemental information). Inter-observer variability of echocardiography was 7.6 %.

### cMRI analysis

**Fig. 4:**
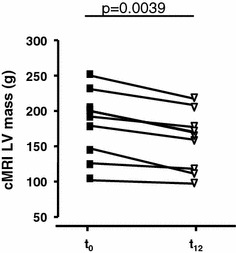
MRI changes during the study period left ventricular (LV) myocardial mass assessed by cardiac MRI in patients with TTR amyloidosis before (*t*_0_) and after 12 months (*t*_12_) of green tea consumption

Clinical characteristics of the subgroups of patients assessed by cMRI (*n* = 9) did not differ from the whole patient cohort (Table 1). We observed a significant average decline of LV myocardial mass by 12.5 % (Fig. 4). In none of the patients an increase of LV myocardial mass was observed. LV ejection fraction remained unchanged (56.7 ± 4.7 vs. 57.2 ± 3.7 %; ns). Detailed cMRI findings are shown in Table 1S (Supplemental information). Inter-observer variability of MRI was 4.2 %.

## Discussion

This is the first single center observation of myocardial parameters during daily consumption of EGCG as GT or GTE for 1 year in patients with cardiac ATTR amyloidosis. No progression of cardiac wall thickness and mass as indicators of ATTR amyloid deposition was observed in these patients during the 1-year observation period. No serious adverse effects were reported.

EGCG as the most abundant catechin in GT has been claimed to prevent all-cause cardiovascular morbidity and mortality [32]. These effects might in part be caused by its lipid lowering effects [33] as observed in all patients of the present study. There are reports indicating that GT or oral supplements of GTE marketed for weight loss may cause serious side effects including acute liver toxicity [34]. In our patients hepatic function remained unchanged during the observation period.

Orthotopic liver transplantation is currently the only available causative treatment of familial amyloid polyneuropathy and/or cardiomyopathy as it eliminates the production of the variant amyloid precursor protein [35] and induces a marked regression (or even disappearance) of amyloid in abdominal fat [36]. In some patients with pre-existing cardiac involvement, progression of amyloid apposition was not halted due to continued deposition of amyloid aggregates from normal TTR aggregates [37–39]. No therapeutic effect of liver transplantation has been observed in SSA patients.

Laboratory experiments have shown that EGCG binds to recombinant wild-type as well as variant TTR tetramers at three sites different from the thyroxine binding site [15, 40, 41]. A stabilizing effect adding to that of the thyroid hormone was observed. Using a transgenic mice model Ferreira et al. [17] demonstrated an inhibitory effect of EGCG on amyloid deposition as well as a degrading effect on preformed amyloid fibrils. The described data, however, were achieved using a 10–100 fold excess of EGCG over TTR, far above the observed plasma concentration (0.5 μM) and applied doses (1 mmol) of EGCG in this trial. However, using 100 nM fibrillar α-synucleine Bieschke et al. [16] found significant aggregate-bound EGCG at concentrations between 20 nM and 1 μM (apparent dissociation constant 0.1 μM). Using isothermal titration calorimetry (ITC) Ferreira et al. [15] found a dissociation constant of 0.4 μM for the interaction of non-denaturated TTR and EGCG.

Progression of left ventricular wall mass within a time span of 1 year was observed in a different group of untreated patients with mutant ATTR and cardiac involvement [42]. It is well known that LV mass varies within the mutation involved and with age at onset of the disease [43]. Survival of patients with cardiac TTR amyloidosis appears to be much better than survival of patients with light-chain amyloidosis [44, 45]. There is, however, a wide variation of 5-year survival rates for patients with different TTR mutations (30–55 %) and among patients with wild-type TTR amyloidosis (40–75 %) [44–47]. The patients in Benson’s study group [42] were about 12 years younger than the patients of our cohort and included individuals with mutations different from those of our patients. Therefore, the results of Benson’s and our present study may not be comparable in a clean scientific approach. The lack of an appropriate control group will affect final conclusion from the present observation.

Cardiac MRI is the gold standard for cardiac morphology and function due to its high reproducibility and low inter-observer variability [48] and has been used as an endpoint in several interventional studies, either for reduction of LV myocardial mass, e. g. arterial hypertension [49], and Fabry’s disease. Fabry [50] or for quantification of scar size after TASH procedure in hypertrophic cardiomyopathy [51]. In the present cohort the reduction of LV myocardial mass by cMRI was more pronounced than the blinded inter-observer variability, but appears to be slow, as observed with enzyme replacement therapy in patients with Fabry’s disease [52–54]. LV wall mass was found unchanged as assessed by echocardiography, possibly due to methodological limitations and limited imaging quality in some patients. However, improved mitral annulus velocity was observed as the most accurate diastolic measure to detect early left ventricular dysfunction in patients with AL amyloidosis [55] and to differentiate restrictive cardiomyopathy from pericarditis constrictiva [56]. In comparison with our echocardiographic data the absolute values of LV myocardial mass were much lower in the present study when assessed by cMRI in comparison with echocardiography. This reflects a well-known phenomenon [57] that was recently shown in a report of patients with hereditary amyloidosis [42, 52]. A possible explanation could be the inclusion of right ventricular trabecula in the measurement of the basal septal wall by echocardiography, especially in patients with massive hypertrophy.

This study was initiated to reproduce Hunstein’s in vivo observation in ATTR cardiomyopathy patients. A decrease of about 25 % of left ventricular wall thickness as reported by Mereles et al. [19] was observed in none of the present patients. As Hunstein suffered from AL amyloidosis and had chemotherapy before he started his tea experiment, the clinical courses of the two different types of disease are not comparable.

Tafamidis, a novel specific inhibitor of TTR tetramer dissociation has been evaluated in a prospective placebo-controlled phase II/III clinical trial in FAP patients and found to reduce the progression of polyneuropathy in the majority of patients (http://www.ema.europa.eu/ema/index.jsp?curl=pages/medicines/human/medicines/002294/human_med_001498.jsp&mid=WC0b01ac058001d124). The binding sites of EGCG have been described to be different from those of known inhibitors for TTR tetramer dissociation [15]. We conclude that due to different modes of interaction, a combined treatment with GT/GTE and tafamidis may complement each other and increase the observed beneficial effects against the major problems of ATTR amyloidosis patients.

Plasma levels of EGCG 2 h after intake of 150 mg EGCG ranged from 44 to 840 μg/L. The individual plasma level of EGCG may be affected by the method of tea preparation, the instability under physiologic conditions, differential gastrointestinal absorption, and the modification by hepatic enzymes [58]. Although we asked our patients to follow a protocol of GT preparation and to use GTE from a single manufacturer, significant uncontrollable modifiers of EGCG bioavailability appear to remain. The endpoint of our study, i.e. change of LV myocardial mass, may be affected by components of GT and GTE other than EGCG bioavailability. We assume that this and a still unknown variability of the interindividual of EGCG might have influence on the observed treatment effects. Adjustment of the individual EGCG plasma levels by appropriate variation of dose or by supporting an increased intestinal EGCG uptake by additional consumption of piperine and ascorbic acid [59] could help ameliorate the effect of treatment. The daily intake dose of EGCG was restricted by the initial design of this study, i.e. to reproduce Hunstein’s in vivo experiment in ATTR cardiomyopathy patients. The lack of an appropriate control collective taking placebo is due to the small number of available patients with ATTR cardiomyopathy in this country. Furthermore, the rather widespread knowledge of the results of Hunstein’s experiment among affected patients resulted in a very low acceptance to renounce on the use of a possible beneficial effect of GT/GTE available to everybody. For ATTR cardiomyopathy being an orphan disease we also were not able to create a control group from safe retrospective data.

GT has been proven beneficial by consumption for several thousands of years by many millions of individuals and is part of the daily routine in the Far East without any reported side-effects. Therefore, its use may be of interest for FAC patients and those at age beyond 65 years with no access to heart transplantation. Randomized, placebo-controlled investigations with longer study periods and larger samples of patients should confirm the results of this observation, determine the optimal dosage of GT/GTE for treatment, and identify undesirable side effects.

## Electronic supplementary material

Below is the link to the electronic supplementary material.

Suppl. Table 1 Data are absolute values and mean ± SEM. mBMI modified body mass index (BMI [kg/m²] ×  serum albumin [g/L] [60]; *IVS* interventricular septum thickness, *LV* left ventricular, *EDD* enddiastolic diameter, *ESV* endsystolic diameter, *EF* ejection fraction, *MAPSE* mitral annular plane systolic excursion, *TAPSE* tricuspid annular plane systolic excursion, *MASV* mitral annular systolic velocity of pulsed-wave Doppler tissue imaging, *EDV* enddiastolic volume, *ESV* endsystolic volume, *SV* stroke volume, *eGFR* estimated glomerular filtration rate, nd not determined. (XLS 43 kb)
